# Genetic Profiling of the Full-Length *tprK* Gene in Patients with Primary and Secondary Syphilis

**DOI:** 10.1128/spectrum.04931-22

**Published:** 2023-04-10

**Authors:** Dan Liu, Li-Li Liu, Xin-Qi Zheng, Rui Chen, Li-Rong Lin, Tian-Ci Yang, Man-Li Tong

**Affiliations:** a Center of Clinical Laboratory, Zhongshan Hospital, School of Medicine, Xiamen University, Xiamen, China; b Institute of Infectious Disease, School of Medicine, Xiamen University, Xiamen, China; Nevada State Public Health Laboratory

**Keywords:** *Treponema pallidum*, full-length *tprK* variants, sequence analysis, syphilis

## Abstract

TprK antigenic variation is acknowledged as an important strategy developed by Treponema pallidum to achieve immune evasion. Previous studies applied short-read sequencing to explore *tprK* gene sequence diversity in clinical samples; however, due to the limitations of short-read sequencing, it was difficult to determine the linkage between the seven V regions, and crucial information about full-length *tprK* variants was lost. Although two recent studies explored complete *tprK* gene profiles in natural human syphilis infection, there are still too few profiled full-length *tprK* variants among clinical T. pallidum isolates to fully understand the characteristics of TprK coding diversity. Here, Pacific Biosciences (PacBio) long-read sequencing was applied to examine the diversity of full-length *tprK* variants in 21 clinical T. pallidum isolates from 11 patients with primary syphilis and 10 patients with secondary syphilis. A total of 398 high-confidence full-length sequences, which presented remarkable sequence heterogeneity, were found. However, these full-length *tprK* variants exhibited limited variation in length and GC content, showing 24 length types and average GC content of 51.5 ± 0.42% and 51.6 ± 0.26% for primary and secondary syphilis samples, respectively. Additionally, the combined patterns of mutated V regions generating new *tprK* variants were obviously different in primary and secondary syphilis samples. The diversity of *tprK* gene sequences in primary syphilis samples may represent the underlying variability of the bacterium; conversely, the variability of the *tprK* gene in secondary syphilis samples may more accurately reflect how T. pallidum escapes host immune clearance. These data highlight the *tprK* gene as an important coding gene that shows conflicting genetic characteristics but underlies the persistence of spirochete infection.

**IMPORTANCE** The resurgence of syphilis in both low- and high-income countries has attracted attention, and persistent infection by the pathogen has long been a research focus. The *tprK* gene, encoding the hypervariable outer membrane protein, is thought to be responsible for pathogen immune evasion and persistent infection. Here, PacBio long-read sequencing was applied to examine the diversity of full-length *tprK* variants in 21 clinical T. pallidum isolates from 11 patients with primary syphilis and 10 patients with secondary syphilis. The results showed that the sequences of the *tprK* gene were remarkably heterogeneous; however, the sequences presented limited variation in length and GC content. The investigation of the combined patterns of the V regions allowed us to gain insight into the features of the *tprK* gene generating new variants at different clinical stages. The findings of this study will be helpful for further exploration of the pathogenesis of syphilis.

## INTRODUCTION

Syphilis, which is caused by the spirochete Treponema pallidum, is a chronic sexually transmitted infection (1, 2). Its explosive resurgence in many countries, particularly among men who have sex with men (MSM) and persons living with HIV, has sounded alarm bells (3, 4). The persistence of the pathogen for the lifetime of an infected individual is the greatest threat to the patient's quality of life (5); however, the mechanism underlying persistent infection is not fully understood. A generally accepted view is that antigen variation allows T. pallidum to escape host immune clearance (6, 7). The rare surface-exposed outer membrane proteins of the pathogen have attracted much attention. The paralogous families of T. pallidum repeat proteins (Tprs) have been identified as belonging to the repertoire of spirochete outer membrane proteins (8). TprK, a member of the Tpr family, not only is thought to be a principal candidate vaccinogen but also is considered to represent an important immune evasion strategy developed by T. pallidum (9–12).

The protein-coding *tprK* gene harbors seven V regions (V1 to V7), each of which is flanked by highly conserved domains (13). Based on a predicted TprK structure, each V region was shown to be exposed as a loop on the surface, and the V regions were found to be antibody targets in experimental syphilis infection (14, 15). The generation of V region diversity was driven by nonreciprocal segmental gene conversion. This gene conversion could create more than 1 million new V sequences and result in significant intra- and interstrain diversity of the *tprK* gene (13). In response to host immune selection, the *tprK* gene showed remarkable accumulation of diversity (16, 17). This finding indicates that understanding the variation of TprK is critical for deciphering the mechanism of lifelong spirochete infection (18, 19).

Recent approaches aimed at understanding TprK antigenic variation have turned to short-read sequencing to push the field forward (19–21). Researchers discovered V sequences on a large scale and gained insight into the diversity of the profiles of the seven V regions of the *tprK* gene. However, due to the limitations imposed by short-read sequencing, these studies had difficulty resolving the linkage between V regions, resulting in the loss of important information about the profiles of full-length *tprK* variants (19–21).

Two recent studies by Addetia et al. applied third-generation (Pacific Biosciences [PacBio]) sequencing to directly profile full-length *tprK* variants (22, 23). In contrast to the redundancy of the V sequences found at the interstrain level (19), none of the full-length *tprK* variants was shared between the analyzed samples. Given the extraordinary diversity present in the *tprK* gene, there are still too few profiled full-length *tprK* variants among clinical T. pallidum isolates to fully understand the characteristics of TprK coding diversity (23). Furthermore, the two studies did not investigate the combinations of the seven V regions in single full-length *tprK* variants, which could provide important information about the generation of a new *tprK* variant.

In this study, we applied PacBio long-read sequencing to explore the diversity of full-length *tprK* variants in 21 clinical T. pallidum isolates from 11 patients with primary syphilis and 10 patients with secondary syphilis. Deep profiling of the diversity of the full-length *tprK* gene and exploration of the linkage between V regions would help us to better understand the characteristic variation of the *tprK* gene at different clinical stages. Obtaining high-confidence full-length *tprK* variants among clinical T. pallidum isolates would provide a critical foundation for developing further serological studies to characterize the anti-TprK immune response.

## RESULTS

### Long-read sequencing data for the *tprK* gene from 28 syphilis samples.

**FIG 1:**
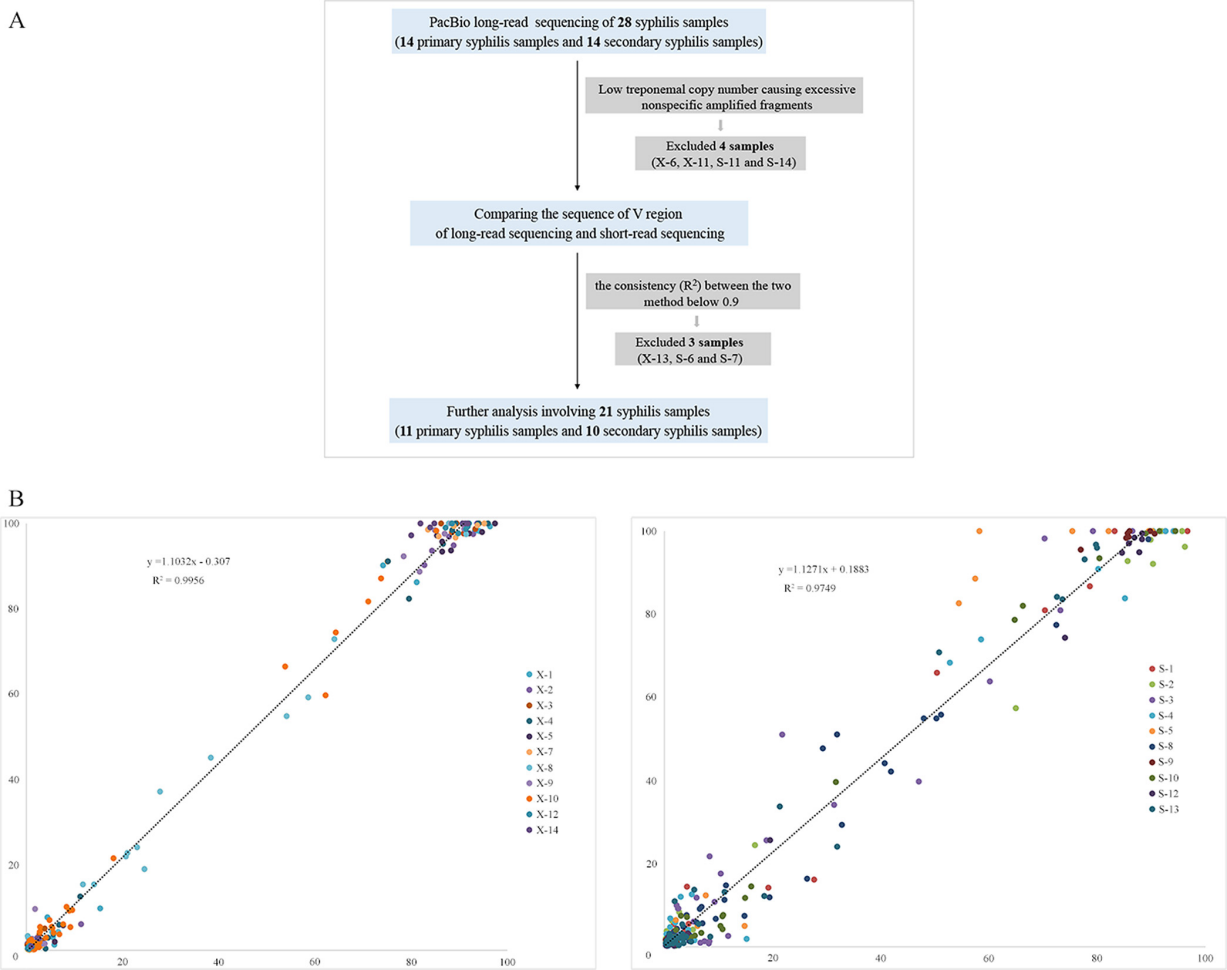
Sample filtering strategy. (A) Flowchart of the strategy. (B) Comparison of long-read and short-read sequencing of the V sequences from 11 primary syphilis samples and 10 secondary syphilis samples.

Long-read sequencing was performed to analyze the full-length *tprK* genes of 28 syphilis samples. This analysis resulted in an average of 4,986 amplicon sequences for the 28 samples (range, 1,086 to 7,602 reads); the average read length was 1,587 bp, with a median read length of 1,590 bp. The detailed read data are shown in Table S2 in the supplemental material. Next, we applied the quality-filtering strategy to obtain high-confidence full-length *tprK* sequences for the 28 samples (Fig. 1A). Four samples with poor sequencing quality (S-11, S-14, X-6, and X-13) were excluded because of excessive nonspecific amplified fragments. Additionally, to evaluate the quality of our long-read sequencing results, we compared the sequences of the V regions obtained from these high-confidence sequences to those obtained from short-read sequencing in previous studies (19). This comparison showed that the *R*^2^ values for consistency of the V sequences between the short reads and the filtered long reads ranged from 0.8821 to 0.993 for primary syphilis samples and from 0.8657 to 0.9976 for secondary syphilis samples. We included only the 21 samples (11 primary syphilis samples and 10 secondary syphilis samples) with consistency values above 0.9 in the final analysis. The comparison of the two sets of sequencing data showed high consistency (*R*^2^ values of 0.9956 and 0.9749 for primary syphilis samples and secondary syphilis samples, respectively) (Fig. 1B).

### Remarkable diversity of full-length *tprK* variants in primary syphilis samples and secondary syphilis samples.

**FIG 2:**
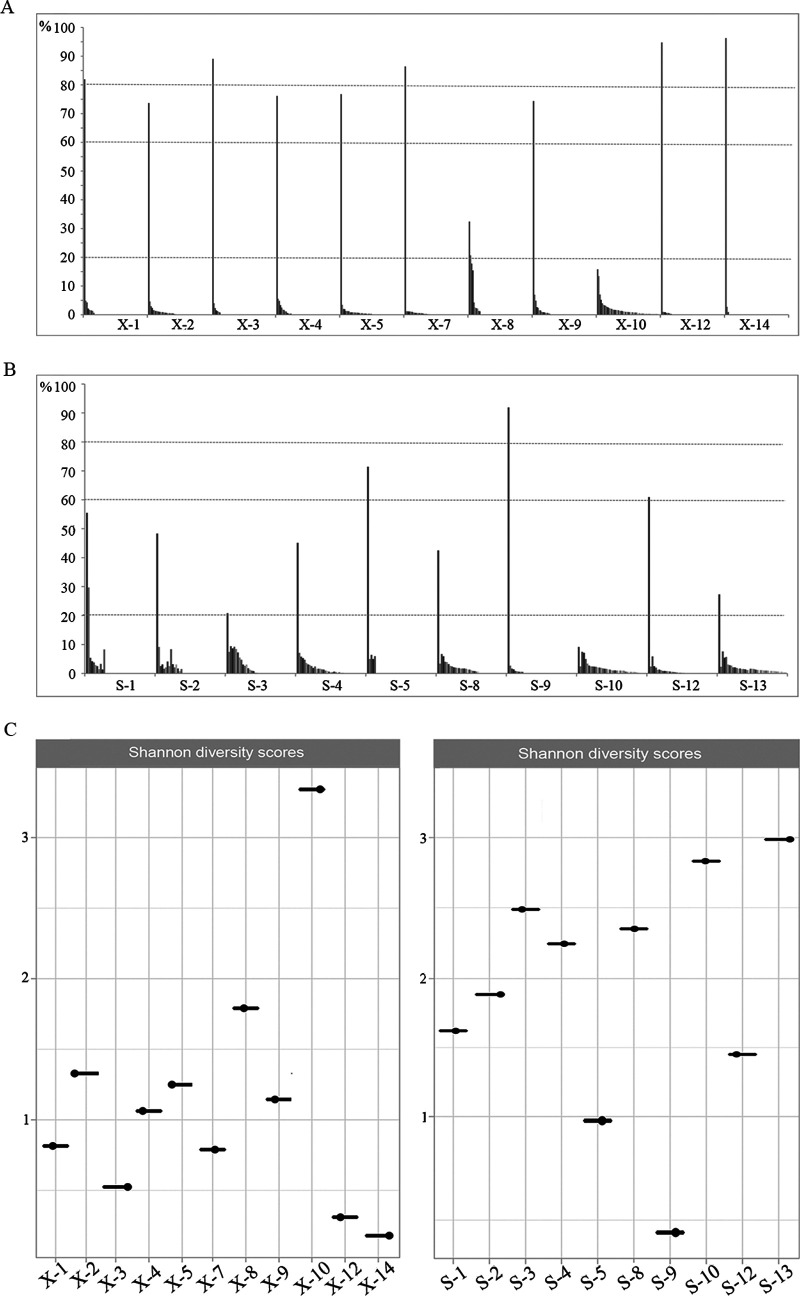
Diversity of full-length *tprK* variants in primary syphilis samples and secondary syphilis samples. (A) Proportional distribution of the distinct full-length *tprK* variants obtained from primary syphilis samples. (B) Proportional distribution of the distinct full-length *tprK* variants obtained from secondary syphilis samples. (C) Shannon diversity measures of the full-length *tprK* variants from primary and secondary syphilis samples.

Using the long-read data, we identified a total of 193 *tprK* variants across 11 primary syphilis samples and 205 variants across 10 secondary syphilis samples (see Table S3). We next found that the distribution of the variants in most primary syphilis samples was dominated by one variant with a frequency of >60% (Fig. 2A). In contrast, the distribution of the variants in most of the secondary syphilis samples was dominated by one variant with a frequency of <60% (Fig. 2B). As shown in Fig. 2, the frequencies of other *tprK* variants found across primary syphilis samples were generally as low as 5% or lower. While this corresponded to the lower frequency of the dominant variant across secondary syphilis samples, some of the minor variants were distributed at frequencies of 5% to 20%. Then, we applied Shannon diversity scores to compare the diversity of the *tprK* variants at the two different stages. The scores for each sample are shown in Fig. 2C. The full-length *tprK* variants from secondary syphilis samples were more diverse than those from primary syphilis samples (*P = *0.035). Additionally, when we aligned all 398 full-length *tprK* variants, it was surprisingly found that none of them was shared across these 21 samples. The variants in each sample appeared to form an independent population.

### Limited variation in the length and GC content of full-length *tprK* variants.

**FIG 3:**
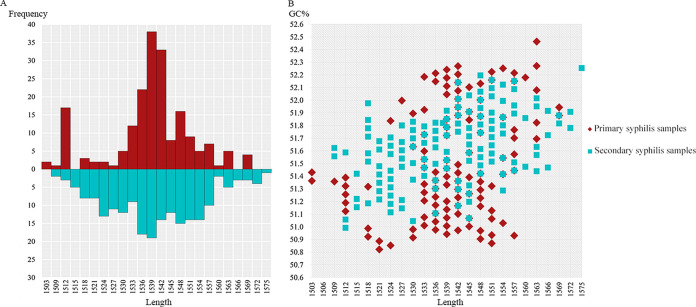
Variation in the length and GC content of full-length *tprK* variants. (A) Distribution of the length of *tprK* variants obtained from primary and secondary syphilis samples. (B) Scatterplot of the length and GC content of *tprK* variants from primary and secondary syphilis samples.

Although the *tprK* variants identified across these 21 samples presented remarkable diversity, the length range of these variants was clearly limited. The lengths of the variants ranged from 1,503 bp to 1,575 bp, including only 24 length types. The distribution of these lengths exhibited a nearly normal distribution, regardless of whether the samples were from primary syphilis or secondary syphilis (Fig. 3A). However, it is worth noting that the length types of the variants in primary syphilis samples presented a more concentrated distribution, centered around a length of 1,539 bp. In contrast, the distribution of secondary syphilis samples was relatively scattered. Subsequently, we calculated the GC content of each full-length *tprK* variant and found that the GC content of the *tprK* variants from primary and secondary syphilis samples showed only slight differences, with average GC content of 51.5 ± 0.42% and 51.6 ± 0.26%, respectively (Fig. 3B). The relationship between GC content and length across the two groups was also analyzed. There was a weak positive correlation between GC content and length in primary syphilis samples (*r* = 0.242, *P < *0.05) and a moderate positive correlation in secondary syphilis samples (*r* = 0.496, *P < *0.05).

### Combined patterns of the seven V regions in full-length *tprK* variants.

**FIG 4:**
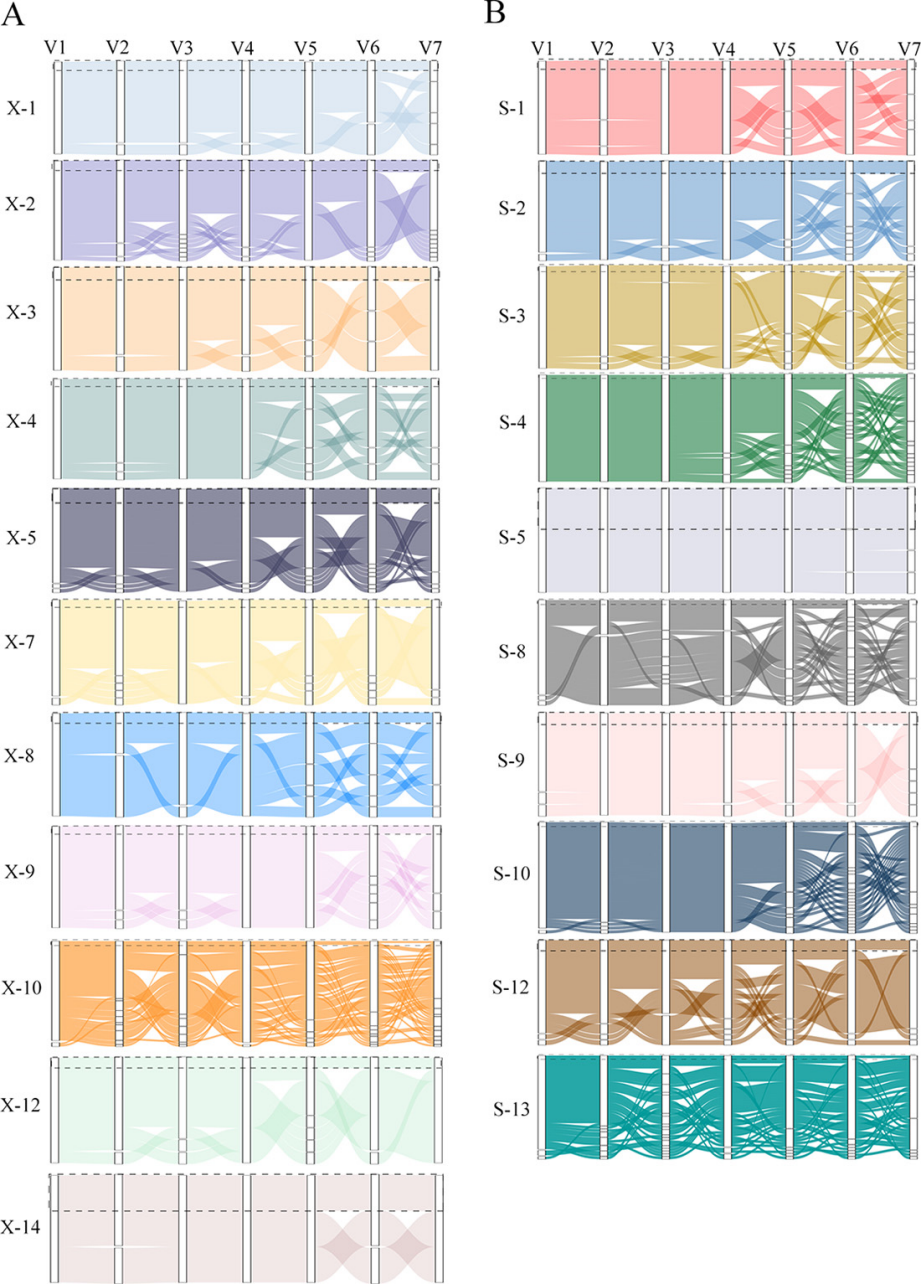
Combined patterns of the seven V regions in full-length *tprK* variants. (A) Primary syphilis samples. (B) Secondary syphilis samples. The sequence usage of the seven V regions in full-length *tprK* variants is represented by the area of the block in each column. The predominant full-length *tprK* variant in each sample is enclosed in a dashed box.

Due to the limitations of short-read sequencing, the V regions obtained by this approach could not be assembled into a full-length *tprK* variant. In this study, high-confidence full-length *tprK* variants were obtained using long-read sequencing, and the features of the seven V regions in the full-length patterns could be further explored. The V sequences of the predominant full-length *tprK* variants of most samples were the sequences with the greatest proportions in the short-sequencing data, except for X-10 and S-3. For sample X-10, the V3 region sequence of the predominant full-length *tprK* variant was not consistent with the greatest proportion of V3 sequences in the short-read sequencing data. This phenomenon occurred in both the V3 and V7 regions in S-3. Further inspection revealed that, in these two samples, the frequency of the predominant full-length *tprK* variant was less than approximately 20%, which was lower than that in the other samples (Fig. 4).

Next, how each V variant assembles into a full-length *tprK* variant, creating the rich diversity of the *tprK* gene, was investigated. In the plots of full-length *tprK* variants, we found that most full-length *tprK* variants in each primary syphilis sample differed from the predominant sequence by only one V region, especially in samples containing a high-frequency full-length *tprK* variant (Fig. 4A). It was rare to find more than two mutant V regions concurrently in the minor full-length *tprK* variant, compared to the predominant sequence. Consequently, sequences within individual samples showed a high degree of similarity. However, as shown in Fig. 4A, the combined pattern of the seven V regions in samples X-8 and X-10 was distinctly different from that described above. More than two mutant V regions in a single full-length *tprK* sequence were frequently observed in these two samples. This scenario became more common across secondary syphilis samples. An increasing number of mutant V regions simultaneously appeared in one minor *tprK* sequence, compared to the predominant sequence (Fig. 4B). Therefore, this increased the diversity of the sequences in secondary syphilis samples. Of course, there were also exceptions, such as samples S-5 and S-9, in which the frequency of the predominant *tprK* variants was relatively high and most of the full-length *tprK* variants had only a single variable V region.

## DISCUSSION

Our previous studies provided a snapshot of the diversity of *tprK* gene sequences in patients with primary and secondary syphilis, based on short-read sequencing (19, 20). Here, we applied long-read sequencing to further investigate the diversity of the full-length profiles of *tprK*. With our filtering criteria, a total of 398 full-length *tprK* sequences were obtained. Based on the lexicon of the seven V regions of the *tprK* gene obtained in our previous studies (19), we found that the V sequences obtained via the two methods showed high consistency, suggesting that the full-length *tprK* variants obtained in this study were of high confidence. When the distribution of the full-length *tprK* variants in each given sample was analyzed, the distribution trend was similar to that we found previously (19), which was also seen in samples from Italy in the study by Addetia et al. (23). This result indicated that the distribution of the *tprK* variants in the primary and secondary syphilis samples was not affected by the geographic area; instead, it might be a genetic characteristic of primary and secondary syphilis.

The lengths of coding sequences are under both functional and structural constraints (24, 25). Low diversity was found among the lengths of the *tprK* variants, and the distribution of these lengths presented a nearly normal distribution between the samples at two different stages, which might indicate that, although TprK is highly variable, the variation is regulated by a strict gene conversion mechanism to maintain structural stability for protein function. Of course, future research is needed to confirm this hypothesis. In addition, the GC content is a factor that constrains the length of the coding sequence in most organisms (26). Therefore, we further explored the relationship between GC content and length among the *tprK* variants and found a positive correlation between GC content and length, indicating that, in addition to functional and structural constraints, GC content is another factor that constrains the length of *tprK* variants. Moreover, the GC content has been reported to be the most prominent property showing strong correlations with recombination in bacteria (27, 28). In this study, the average GC content of the *tprK* variants in secondary syphilis samples was only slightly higher than that in primary syphilis samples, suggesting that *tprK* gene conversion might not be G/C biased. This finding supported the results of the study by Lin et al., which showed no significant differences in GC content in highly variable V6 sequences (21). Generally, the limited variations in the lengths and GC contents of the full-length *tprK* variants seemed to conflict with the highly variable features of the *tprK* gene but were not unreasonable.

The advantages provided by long reads made the large-scale exploration of the combinations of the seven V regions in a single *tprK* variant possible. By aligning the full-length *tprK* variants of each sample and examining the mutant V regions (taking the predominant sequence as a reference), we found that most full-length *tprK* variants of primary syphilis samples contained only one mutant V region. This pattern of variant emergence should greatly reduce the potential diversity of the *tprK* gene, causing the *tprK* variants within a sample to be highly similar. Conversely, the pattern of variant emergence in secondary syphilis samples was more in line with our expectation that different mutant V regions would appear together in a single full-length *tprK* variant. Different combinations of mutant V regions would significantly increase the diversity of the *tprK* gene, in turn reducing the similarity between sequences and resulting in a greater likelihood of inducing TprK antigenic variation to facilitate secondary syphilis development (18). As reported in a recent study in which the *tprK* gene was shown to maintain a low but detectable basal rate of variation in the complete absence of immune pressure (21), the profile of the *tprK* variants in primary syphilis samples seemed more similar to that observed in the absence of immune pressure, showing basal variability. The *tprK* gene then accumulates more diversity on top of its own basal variation to facilitate disease development, as shown by the profile of the *tprK* gene in secondary syphilis samples. The variability of the *tprK* gene in secondary syphilis samples would more accurately reflect how T. pallidum escapes host immune clearance (18).

Notably, the full-length *tprK* variants were not found to be shared among the 21 samples, as reported by Addetia et al. (23). A previous study also demonstrated that an initially infecting isolate and a reinfecting isolate did not share any full-length *tprK* variants (22). Even full-length *tprK* variants were not shared in recurrent cases (29). Instead, the full-length *tprK* variants within the strain were more likely to cluster together (22, 23, 29). This result may indicate that each *tprK* variant is unique, although they were generated from the same repertoire of genome donor sites by nonreciprocal segmental gene conversion (13, 23). Therefore, it has become more important to clarify the function of TprK. It would be helpful to understand the reasons for the high variability of the *tprK* gene.

However, it must be noted that the total number of full-length *tprK* variants discovered in our study was substantially lower than that reported in studies by Addetia et al. (22, 23). The difference in counts between the two studies was mainly due to a large difference in the number of *tprK* sequences in secondary syphilis samples. This could be the consequence of the fewer PacBio reads obtained in our study. Because primary syphilis samples with more *tprK* variants were present at lower frequencies (<0.2%), PacBio reads from both studies could not confidently call these *tprK* variants. However, secondary syphilis samples had more *tprK* variants present at frequencies of 5 to 10%, and fewer PacBio reads may increase the chances of losing information about these *tprK* variants. Nevertheless, our results indicated that long-read sequencing was remarkably consistent with short-read sequencing for detecting TprK variants. In addition, we did not further explore the structures of the full-length *tprK* variants to determine whether there were structural changes in the *tprK* variants. Future work should attempt to follow a “structure-to-pathogenesis” approach to map the surface topology of TprK within the context of syphilitic infection, based on a more complete profile of the *tprK* gene.

Overall, the results showed that the *tprK* gene not only presented remarkable sequence heterogeneity but also had relatively conserved properties. The combined patterns of mutated V regions generating new *tprK* variants in primary and secondary syphilis samples were obviously different. The findings of this study will contribute to further exploration of the pathogenesis of syphilis.

## MATERIALS AND METHODS

### Ethics statement.

The subjects included in this study were adults, and all of the subjects provided written informed consent in accordance with the institutional guidelines prior to the study. This study was approved by the Institutional Ethics Committee of Zhongshan Hospital, School of Medicine, Xiamen University, and complied with national legislation and the Declaration of Helsinki guidelines.

### Amplification and sequencing of *tprK*.

DNA samples from previous studies that had been extracted from 14 primary syphilis samples (genital swab samples) and 14 secondary syphilis samples (lesion biopsy samples) (19, 20) were directly used for the PCR amplification of *tprK*. The detailed characteristics of the samples were described in the previous study (19). High-fidelity PrimeSTAR GXL DNA polymerase (TaKaRa Bio, Inc., Beijing, China) was used in the PCR system to ensure the accuracy of amplification. PCR was conducted with *tprK*-specific primers appended to 16-bp PacBio barcodes (see Table S1 in the supplemental material) under the following conditions: 98°C for 2 min, 35 cycles of 98°C for 10 s, 62°C for 15 s, and 68°C for 2 min, and a final elongation at 68°C for 5 min. The resulting *tprK* amplicon was purified using 0.6 volume of AMPure XP beads (Beckman Coulter, Inc., Brea, CA, USA). Library construction and sequencing on a Sequel I single-molecule real-time (SMRT) Cell 1M system, with a 10-h movie, were completed by Beijing Novogene Bioinformatics Technology.

### Processing and filtering of PacBio reads.

The output data were filtered and processed using SMRT Link v4.0, and PacBio subreads generated for each amplicon were converted to circular consensus (CCS) reads using CCS v3.0.0. Reads with a consensus base call confidence of <99% were excluded. Based on the published custom Python/R scripts available on GitHub (22, 23), scripts were modified to run on our data in-house. Briefly, the retained PacBio reads with lengths between 1,400 and 1,800 bp were trimmed of PCR primers by using the DADA2 preprocessing pipeline and were further denoised by using RAD. The V region sequences previously obtained by short-read sequencing were used for quality checking of the full-length *tprK* sequences (19). A receivable full-length *tprK* sequence had to satisfy the following conditions: (i) each V region must be supported by ≥5 reads from short-read sequencing, and (ii) it must not contain a stop codon or frameshift. The detailed bioinformatic pipeline used in the study is also available on GitHub (https://github.com/lauisld/Amoy-research-lab). Shannon diversity scores for each sample were calculated as described previously (23). Diversity scores for strains stratified by clinical stage were assessed using the Wilcoxon rank-sum test. Two-sided *P* values of <0.05 were considered statistically significant.

### Data availability.

PacBio reads obtained from the *tprK* sequencing of the samples in this study are available under NCBI BioProject accession number PRJNA902089.
