# Supplementary material for: Genetic Profiling of the Full-Length tprK Gene in Patients with Primary and Secondary Syphilis
Source: Microbiol Spectr. 2023 Apr 10;11(3):e04931-22. doi: 10.1128/spectrum.04931-22 (PMC10269439; doi:10.1128/spectrum.04931-22)
Supplement: Supplemental file 1 — Tables S1 and S2. Download spectrum.04931-22-s0001.pdf, PDF file, 0.2 MB [file spectrum.04931-22-s0001.pdf]

SUPPLEMENTAL MATERIAL

TABLE S1. PacBio barcoded *tprK* primers used in this study

TABLE S2. The long-read sequencing data of the *tprK* gene in the 28 clinical syphilis samples

TABLE S3. The obtained full-length *tprK* sequences in the study. (A) from primary syphilis samples (B) from secondary syphilis samples

Table S1. PacBio barcoded *tprK* primers used in this study

Primer Name	Sequence (5' -> 3')
tprK-F_bc X-1	**TAGTGTGCGACTCTGA**ACCGGGCATGAATTTTCTTT
tprK-F_bc X-2	**TCGTCATACGCTCTAG**ACCGGGCATGAATTTTCTTT
tprK-F_bc X-3	**GCGCGCGCACTCTCTG**ACCGGGCATGAATTTTCTTT
tprK-F_bc X-4	**TGTGCTCTCTACACAG**ACCGGGCATGAATTTTCTTT
tprK-F_bc X-5	**TATACGAGATACGTGA**ACCGGGCATGAATTTTCTTT
tprK-F_bc X-6	**GCTCGACTGTGAGAGA**ACCGGGCATGAATTTTCTTT
tprK-F_bc X-7	**CGCTAGAGATCTGCTA**ACCGGGCATGAATTTTCTTT
tprK-F_bc X-8	**TGTACGCTCTCTATAT**ACCGGGCATGAATTTTCTTT
tprK-F_bc X-9	**AGCACGTGTGTCGACA**ACCGGGCATGAATTTTCTTT
tprK-F_bc X-10	**TCTCACTGATAGCGTG**ACCGGGCATGAATTTTCTTT
tprK-F_bc X-11	**TAGCGTGAGAGTGTCG**ACCGGGCATGAATTTTCTTT
tprK-F_bc X-12	**ACACGTGTGCTCTCTC**ACCGGGCATGAATTTTCTTT
tprK-F_bc X-13	**TCTATGAGCACTCTCG**ACCGGGCATGAATTTTCTTT
tprK-F_bc X-14	**TCTCACGAGAGCGCAC**ACCGGGCATGAATTTTCTTT
tprK-R_bc X-1	**TCAGAGTCGCACACTA**CCATACATCCCTACCAAATCA
tprK-R_bc X-2	**CTAGAGCGTATGACGA**CCATACATCCCTACCAAATCA
tprK-R_bc X-3	**CAGAGAGTGCGCGCGC**CCATACATCCCTACCAAATCA
tprK-R_bc X-4	**CTGTGTAGAGAGCACA**CCATACATCCCTACCAAATCA
tprK-R_bc X-5	**TCACGTATCTCGTATA**CCATACATCCCTACCAAATCA
tprK-R_bc X-6	**TCTCTCACAGTCGAGC**CCATACATCCCTACCAAATCA
tprK-R_bc X-7	**TAGCAGATCTCTAGCG**CCATACATCCCTACCAAATCA
tprK-R_bc X-8	**ATATAGAGAGCGTACA**CCATACATCCCTACCAAATCA
tprK-R_bc X-9	**TGTCGACACACGTGCT**CCATACATCCCTACCAAATCA
tprK-R_bc X-10	**CACGCTATCAGTGAGA**CCATACATCCCTACCAAATCA
tprK-R_bc X-11	**CGACACTCTCACGCTA**CCATACATCCCTACCAAATCA
tprK-R_bc X-12	**GAGAGAGCACACGTGT**CCATACATCCCTACCAAATCA
tprK-R_bc X-13	**CGAGAGTGCTCATAGA**CCATACATCCCTACCAAATCA
tprK-R_bc X-14	**GTGCGCTCTCGTGAGA**CCATACATCCCTACCAAATCA
tprK-F_bc S-1	**TCATACACACAGATAG**ACCGGGCATGAATTTTCTTT
tprK-F_bc S-2	**TATAGAGCTCTACATA**ACCGGGCATGAATTTTCTTT
tprK-F_bc S-3	**GCGCTCTCTCACATAC**ACCGGGCATGAATTTTCTTT
tprK-F_bc S-4	**CACATATCAGAGTGCG**ACCGGGCATGAATTTTCTTT
tprK-F_bc S-5	**TGACAGTATCACAGTG**ACCGGGCATGAATTTTCTTT
tprK-F_bc S-6	**TGAGTGAGACATATCA**ACCGGGCATGAATTTTCTTT
tprK-F_bc S-7	**TATCTGAGCGCGAGCA**ACCGGGCATGAATTTTCTTT
tprK-F_bc S-8	**TCACGTGCAGATATAG**ACCGGGCATGAATTTTCTTT
tprK-F_bc S-9	**TCTCATGTGTGAGCTA**ACCGGGCATGAATTTTCTTT
tprK-F_bc S-10	**GCGTGTGTCGAGTGTA**ACCGGGCATGAATTTTCTTT
tprK-F_bc S-11	**TCTGTATCTCTATGTG**ACCGGGCATGAATTTTCTTT
tprK-F_bc S-12	**ACACACTCTATCAGAT**ACCGGGCATGAATTTTCTTT
tprK-F_bc S-13	**CGTATACAGTCACGCT**ACCGGGCATGAATTTTCTTT

tprK-F_bc S-14	**CTGTGCTATGTACGCG**ACCGGGCATGAATTTTCTTT
tprK-R_bc S-1	**CTATCTGTGTGTATGA**CCATACATCCCTACCAAATCA
tprK-R_bc S-2	**TATGTAGAGCTCTATA**CCATACATCCCTACCAAATCA
tprK-R_bc S-3	**GTATGTGAGAGAGCGC**CCATACATCCCTACCAAATCA
tprK-R_bc S-4	**CGCACTCTGATATGTG**CCATACATCCCTACCAAATCA
tprK-R_bc S-5	**CACTGTGATACTGTCA**CCATACATCCCTACCAAATCA
tprK-R_bc S-6	**TGATATGTCTCACTCA**CCATACATCCCTACCAAATCA
tprK-R_bc S-7	**TGCTCGCGCTCAGATA**CCATACATCCCTACCAAATCA
tprK-R_bc S-8	**CTATATCTGCACGTGA**CCATACATCCCTACCAAATCA
tprK-R_bc S-9	**TAGCTCACACATGAGA**CCATACATCCCTACCAAATCA
tprK-R_bc S-10	**TACACTCGACACACGC**CCATACATCCCTACCAAATCA
tprK-R_bc S-11	**CACATAGAGATACAGA**CCATACATCCCTACCAAATCA
tprK-R_bc S-12	**ATCTGATAGAGTGTGT**CCATACATCCCTACCAAATCA
tprK-R_bc S-13	**AGCGTGACTGTATACG**CCATACATCCCTACCAAATCA
tprK-R_bc S-14	**CGCGTACATAGCACAG**CCATACATCCCTACCAAATCA

Table S2. The long-read sequencing data of the *tprK* gene in the 28 clinical syphilis samples

Sample	Raw reads	Clean reads	Base (nt)	Avg Len (nt)	Effective %
X-1	4546	4474	7126848	1592	98.42
X-2	7152	7020	11178264	1592	98.15
X-3	5551	5497	8754668	1592	99.03
X-4	4959	4896	7794719	1592	98.73
X-5	7268	7129	11354200	1592	98.09
X-6	6889	6684	10606398	1586	97.02
X-7	8076	7350	11525568	1568	91.01
X-8	1154	1086	1724578	1588	94.11
X-9	3165	3095	4926502	1591	97.79
X-10	5784	5677	9031877	1590	98.15
X-11	6392	6138	9729706	1585	96.03
X-12	5523	5414	8627022	1593	98.03
X-13	1471	1394	2215111	1589	94.77
X-14	1377	1321	2098654	1588	95.93
S-1	2163	2088	3323146	1591	96.53
S-2	6355	6296	10018307	1591	99.07
S-3	8002	7602	12099001	1591	95
S-4	7709	7560	11916631	1576	98.07
S-5	5385	5266	8362915	1588	97.79
S-6	6962	6615	10530164	1591	95.02
S-7	2935	2819	4485336	1591	96.05
S-8	2436	2324	3697159	1590	95.4
S-9	6327	6267	9953501	1588	99.05
S-10	4805	4678	7440165	1590	97.36
S-11	6200	5766	9186670	1593	93
S-12	6480	6418	10112690	1575	99.04
S-13	3389	3317	5267864	1588	97.88
S-14	5472	5420	8535148	1574	99.05
